# Long-Lasting Insecticidal Hammocks for Controlling Forest Malaria: A Community-Based Trial in a Rural Area of Central Vietnam

**DOI:** 10.1371/journal.pone.0007369

**Published:** 2009-10-07

**Authors:** Ngo Duc Thang, Annette Erhart, Niko Speybroeck, Nguyen Xuan Xa, Nguyen Ngoc Thanh, Pham Van Ky, Le Xuan Hung, Le Khanh Thuan, Marc Coosemans, Umberto D'Alessandro

**Affiliations:** 1 National Institute of Malariology, Parasitology and Entomology, Hanoi, Vietnam; 2 Prince Leopold Institute of Tropical Medicine, Antwerp, Belgium; 3 Ecole de santé publique, Université Catholique de Louvain, Bruxelles, Belgium; 4 Provincial Centre for Malariology, Parasitology and Entomology, Ninh Thuan, Vietnam; Walter and Eliza Hall Institute of Medical Research, Australia

## Abstract

**Background:**

In Vietnam, malaria remains a problem in some remote areas located along its international borders and in the central highlands, partly due to the bionomics of the local vector, mainly found in forested areas and less vulnerable to standard control measures. Long Lasting Insecticidal Hammocks (LLIH), a tailored and user-friendly tool for forest workers, may further contribute in reducing the malaria burden. Their effectiveness was tested in a large community-based intervention trial carried out in Ninh Thuan province in Central Vietnam.

**Methods and Findings:**

Thirty villages (population 18,646) were assembled in 20 clusters (1,000 individuals per cluster) that were randomly allocated to either the intervention or control group (no LLIH) after stratification according to the pre-intervention *P. falciparum* antibody prevalence (<30%; ≥30%). LLIH were distributed to the intervention group in December 2004. For the following 2 years, the incidence of clinical malaria and the prevalence of infection were determined by passive case detection at community level and by bi-annual malariometric surveys. A 2-fold larger effect on malaria incidence in the intervention as compared to the control group was observed. Similarly, malaria prevalence decreased more substantially in the intervention (1.6-fold greater reduction) than in the control group. Both for incidence and prevalence, a stronger and earlier effect of the intervention was observed in the high endemicity stratum. The number of malaria cases and infections averted by the intervention overall was estimated at 10.5 per 1,000 persons and 5.6/100 individuals, respectively, for the last half of 2006. In the high endemicity stratum, the impact was much higher, i.e. 29/1000 malaria cases and 15.7 infections/100 individuals averted.

**Conclusions:**

LLIH reduced malaria incidence and prevalence in this remote and forested area of Central Vietnam. As the targets of the newly-launched Global Malaria Action Plan include the 75% reduction of the global malaria cases by 2015 and eventually the elimination/eradication of malaria in the long term, LLIH may represent an additional tool for reaching such objectives, particularly in high endemicity areas where standard control tools have a modest impact, such as in remote and forested areas of Southeast Asia and possibly South America.

**Trial Registration:**

ClinicalTrials.gov NCT00853281

## Introduction

The recently-launched “Global Malaria Action Plan” calls for a malaria-free world, renewing for the first time since the global malaria eradication campaign was abandoned in the early 70's the hope that the elimination and eventually the eradication of malaria is achievable in the long term [1]. As malaria epidemiology varies between countries or even regions, control efforts should be adapted to the local situation. In places where malaria transmission is low to moderate, targeted vector control measures such as indoor residual spraying (IRS) or insecticide-treated bed nets (ITNs) can be used efficiently [1]. Nevertheless, the impact of these interventions may be lower than expected if either the vector is less vulnerable because of its behaviour or the local populations, for geographical, socio-economical or cultural reasons, are less reachable or compliant. Therefore, there is the need of formulating new approaches and designing new tools able to tackle context-specific constraints.

Vietnam has been extremely successful in controlling malaria; in 2000, malaria mortality had decreased since 1991 by 97%; by 2007, only 70,910 malaria cases and 20 malaria deaths were reported in a country of 80 million people, a 93.5% and 99.6% decrease compared to 1991 [2]. Despite these successes, malaria remains an important disease along international borders with Laos and Cambodia and in the central highlands, where about half of all malaria cases and 80% of severe cases and malaria-related deaths occurs [2]–[4]. These areas are remote, forested and populated by ethnic minorities living traditionally on forest related activities. In addition, IRS and ITN have little effect on the main vector *Anophele dirus s.s.*, a sylvatic species highly anthropophylic, with a behaviour characterized by exophagy, exophily and early biting [5]–[6] . New control tools, better adapted to this situation, are needed. In Central Vietnam, where hammock use is common and forest activity has been identified as a strong risk factor for malaria infection [7]–[9], long-lasting insecticidal hammocks (LLIH) may be an additional tool to effectively reduce the malaria burden. Their effectiveness was evaluated within a community-based intervention trial carried out in Ninh Thuan, one of the highest endemic malaria provinces in Vietnam, inhabited by the Ra-glai ethnic group [8]–[9]. Results are reported below.

## Methods

The protocol for this trial and supporting CONSORT checklist are available as supporting information; see Checklist S1 and Protocol S1.

### Ethical considerations

The study protocol, including the procedure for verbal consent that was specified in the document submitted, was approved by the ethical committees of the Institute of Tropical Medicine, Antwerp, Belgium, and the NIMPE, Hanoi, Vietnam, as well as by the Vietnamese Ministry of Health. The fundamental principles of ethics in research on human participants were maintained throughout the study period. The research procedures were disclosed to all participants (community leaders and local authorities were witnesses) at the time of the census, and oral informed consent was sought from them or their legal representatives. It was estimated that the procedure of verbal consent would be sufficient as people living in the study villages could choose on whether or not use the intervention (LLIH). In addition, the study procedures, i.e. the identification of malaria infections at village and health facility level, including the cross-sectional surveys, were within the activities carried out by government authorities for the purpose of malaria control. Nobody was coerced into the study and if individuals wished to withdraw, they were allowed to do so without prejudice.

### Study site & population

**Figure 1:**
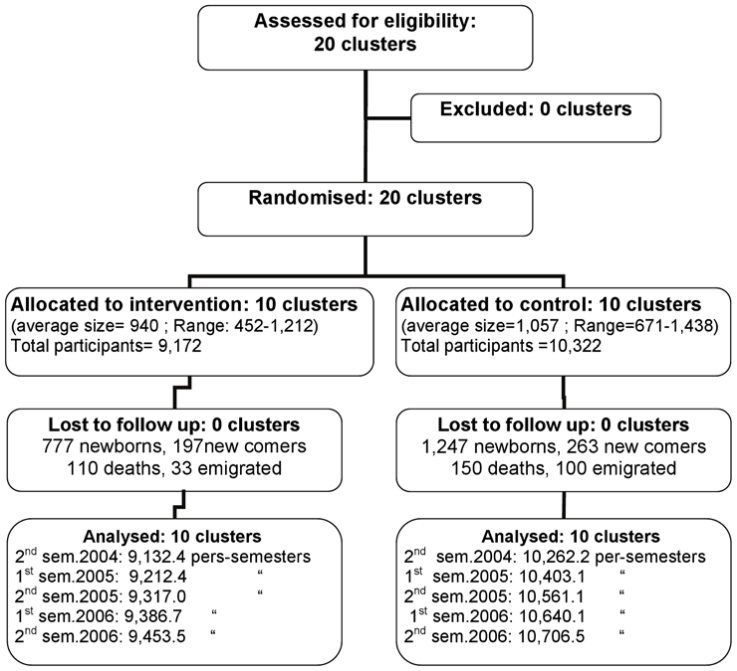
Flowchart of the study.

The study used a cluster randomized design because its main objective was to establish the effectiveness of LLIH at community level, including a potential mass effect, mimicking its implementation in operational conditions. In addition, contamination, i.e. sharing of hammocks by different people within the same community if the randomization had been done at individual level, was a concern. The study was implemented in 30 villages located in 2 districts (25 villages in eight communes of Bac Ai, and five in of two communes in Ninh Son district) of the central southern coastal province of Ninh Thuan (Figure 1), already described elsewhere [9]. Briefly, the population is mainly represented by the Ra-glai ethnic group, a largely impoverished minority living mainly on small scale farming in forest plots (manioc, maize, cashew) and forest products exploitation (bamboo, resin, hunting). Malaria transmission is perennial with two peaks, in June and October, and the two main vectors are *An. dirus sensu stricto* and *Anopheles minimus A*. Between 2001 and 2003, the estimated malaria incidence per year (confirmed cases) in the whole province of Ninh Thuan was between 7.2 and 3.4/1000 [10]–[11].

In March 2004, the whole study population was enumerated (each resident was assigned a unique code number) and information on age, sex, ethnicity, occupation, socio-economic status, forest activity (type and amount), bed net use and other malaria preventive practices was collected. Births, deaths and migrations were registered by village health workers (VHW) and included monthly in the census file [12].

### Sample size calculation and randomization process

The sample size was estimated taking into account the cluster randomized design [13] and assuming a reduction of 30% in malaria prevalence and 33% in malaria incidence (at 5% significance level and 80% power) within 2 years in the intervention as compared to the control group. Ten clusters (1,000 inhabitants each) per study group were necessary, and, within each cluster, a cohort of 160 individuals randomly selected for the bi-annual surveys were included in the study. In each cluster, 60 children 2–9 years old were later added to the cohort following the high prevalence of malaria infection in this age group at the baseline survey (April 2004). Each cluster comprised one to three neighbouring villages, according to their size, to total about 1,000 individuals. Incidence of malaria cases was determined in the total population. Before randomisation, clusters were stratified according to the prevalence of antimalarial antibodies determined in a 2003 survey [8] that showed high variability between villages, with values ranging from 0 to 75%. A “high endemicity” (*P.falciparum* sero-prevalence≥30%) and “low endemicity” (sero-prevalence<30%) strata were defined, resulting in balanced groups within each stratum, i.e. *P.f* seroprevalence of 35.2% (95% CI: 24.9; 47.2) in the control *versus* 42.0% (95% CI 26.0; 60.3) in the intervention group in the high endemicity stratum, and 20.0% (95% CI: 15.1; 26.0) *vs* 19.9% (95% CI: 15.2; 25.5) in the low endemicity stratum. Within each stratum, clusters were assigned a unique number (from 1 to 10) and then randomized to intervention or control following a computer-generated list using EpiInfo v6.04d (CDC, Atlanta; WHO, Geneva 1996). The list was generated and held at the Institute of Tropical Medicine, Antwerp, Belgium.

### Intervention: Long Lasting Insecticidal Hammocks (LLIH)

The Olyset® net (Sumitomo Chemical Co Ltd, Japan), one of the two LLIN recommended by WHOPES at the time the project started [14], consists of high density polyethylene with 2% Permethrin, a synthetic pyrethroid directly incorporated during the net fabrication. The active ingredient, embedded within the fibre and slowly released over time, allows for bioavailability of the insecticide at the surface of the fibre, with a residual effect lasting for three to five years. The hammocks were produced and sewn with Olyset netting in the Binh Dinh Textile Company in Quy Nhon province, Vietnam. Hammocks were made of green nylon (double layered 2.07 m length, 1.03 m width) and two woven ropes (1.5 cm diameter, 3.7 m length). The Olyset net had double the width of the hammock and was sewn for one half of the width onto the back side of the hammock with 10 parallel seams, and the second half was a free flap to be wrapped over when laying inside. In December 2004, at the time of the second survey, 7,000 LLIHs were individually distributed to all residents (≥10 years old) in the intervention clusters, obtaining a 70% coverage of the intervention population. No LLIH was distributed in the control group.

### Assessment of LLIH effectiveness

The effect of the intervention was estimated by monitoring malaria incidence and prevalence through an extensive surveillance system combining bi-annual cross-sectional surveys and passive case detection at village level.

#### Bi-annual cross-sectional surveys

Between April 2004 and December 2006, six biannual cross-sectional surveys (at the beginning (April), and at the end (November) of the rainy season) were carried out. Participants were interviewed on previous malaria symptoms and anti-malarial treatments taken. A clinical examination was carried out by a physician (body temperature and spleen size) and a blood sample was collected for microscopic examination (blood smear) and later serological analysis (filter paper). Suspected malaria cases were presumptively treated with either chloroquine (25 mg/kg over 3 days) or artesunate (7 days) according to the symptoms. Survey participants belonging to the intervention group were asked whether they had received a LLIH and, if yes, how they used it (location: village and/or forest; and time: day/evening/night/never).

#### Passive case detection at community level

The passive detection of malaria cases, carried out over the whole population, started in July 2004 and continued until the end of the study. Patients attending either the Commune Health Centres (CHC) or consulting the VHW were first identified onto the census file. The body temperature and a blood slide to be read later were systematically collected. A rapid diagnostic tests (RDT) was done on all patients who were treated on the basis of its results: *P.falciparum* (including mixed infections) with a full course of artesunate (16 mg/kg) for 7 days, *P.vivax* with chloroquine (25 mg/kg) for 3 days. This information was registered on a pre-coded standardized questionnaire. Quality of case management, blood sampling, and reporting was ensured by monthly supervision meetings by the staff of both the Provincial Centre for Malariology, Parasitology and Entomology (PCMPE) and the District Health Centres (DHC), which insured continuous retraining of the VHWs and the CHC health staff as required.

### Laboratory tests

#### Rapid diagnostic test

Paramax-3™ (Zephyr Biomedicals, India) rapid tests for detecting *P. falciparum*-specific histidine rich protein-2 (Pf HRP-2), *P. vivax* specific lactacte dehydrogenase (pLDH) and a pan malaria-specific pLDH were used, with detailed user's recommendation published elsewhere [12].

#### Microscopic examination

Blood slides were stained with a 3% Giemsa solution for 45 minutes. The number of asexual parasites per 200 white blood cells (WBCs) was counted and parasite densities were computed assuming a mean WBC count of 8,000/µL. Similarly, the density of sexual forms was also determined for each parasite species. A slide was defined as negative if no asexual form was found after counting 1,000 WBCs. Microscopic examination was blinded to patients' identity and location: reading and quality control was performed at the National Institute of Malariology, Parasitology and Entomology in Hanoi. Discrepant results were re-read and agreed upon by a third senior technician.

### Case definition

Patients with malaria symptoms consulting the VHW or attending the CHC were considered as suspected malaria cases. A malaria infection was defined as a positive blood slide with Plasmodium asexual forms, regardless of symptoms and parasite density. Clinical malaria was defined as a patient with fever (body temperature ≥37.5°C), and/or history of fever in the past 48 hours, and a positive blood slide for Plasmodium asexual forms. Recrudescence was defined as clinical malaria occurring within 28 days following the first episode with the same parasite species. Splenomegaly was defined as any palpable spleen, independently of the Hackett classification.

### Data management and statistical analysis

Data were double entered, checked and cleaned using EpiInfo v6.04d (CDC, Atlanta; WHO, Geneva 1996); the analysis was done with STATA 9.0 software (Stata Corp., College Station, TX). Descriptive statistics were used to compute proportions and means, taking into account the survey characteristics (“svy” command in STATA). For PCD data, the total population follow-up time was divided into five consecutive semesters. Malaria incidence rates per 6-month period were computed by dividing the number of new cases in a given semester by the corresponding total person-semester at risk obtained from the demographic follow-up. The latter was obtained by computing for each individual the number of days spent in the study area in relation to the actual length in days of that specific semester. Incidence rates were compared and incidence rate ratios computed using a Poisson survey regression model, allowing for the survey design, with an interaction term fitted between the time and trial variables. Similarly, for the effect on prevalence, a survey logistic model including the interaction term was used. Indeed, as the incidence/prevalence rates before the intervention were substantially different between the 2 study groups (see below), an indirect approach for the estimation of the effect of the intervention was used. The reductions in incidence/prevalence observed across time (consecutive semesters/surveys) in the intervention group were compared with the corresponding ones in the control group, both for the whole study population as well as within each stratum. More specifically, the effect on malaria prevalence across five consecutive surveys (and incidence rates over five semesters) were measured within each study group using odds ratios (OR_ = _odds of infection at survey x/odds of infection at the Dec.04 survey) and incidence rate ratios (IRR = incidence rate at semester x/incidence rate at semester Jul-Dec.04) for the effects of time. The correlation between serial measures of incidence and prevalence was assessed by fitting generalized estimating equation models (“xtgee” command in STATA) that resulted in narrower confidence intervals. Therefore, the simpler and most conservative model (“svy”), taking into account the sampling design, was chosen for the analysis. The difference of effect in prevalence/incidence reductions (in terms of ratio) between intervention and control group was assessed through the point estimate and significance (Wald test p<0.05) of the interaction term. In order to confirm that the observed difference in reduction (prevalence/incidence) in the high stratum (stratum 1) was not due to unbalanced starting points, i.e. higher prevalence group experiencing higher reduction, the analysis was repeated with newly defined strata which were more homogenous in terms of pre-intervention parasite prevalence (parasite rate in the Dec.04 <20% (“new stratum 2”), or ≥20% (“new stratum 1”).

The number of cases averted by the intervention (clinical cases and infections) was estimated for the whole study population as well as per stratum. The expected final incidence rate (last semester 2006) in the control group, if the two groups would have been similar at the start, was calculated by multiplying the final incidence in the intervention group by the interaction term. The number of averted cases was computed by the difference between the expected incidence rate in the control group and the actual incidence rate in the intervention group. A similar approach, using parasite rates instead of incidence rates, was used for estimating the number of malaria infections averted at the last survey.

## Results

In March 2004, the study population included 18,646 individuals; intervention and control groups were comparable for a series of socio-demographic characteristics and bed net use, which was over 90% when considering also untreated bed nets, and over 70% among people sleeping in the forest when considering both ITN alone or ITN and use of traditional hammock (Table 1).

**Table 1:** Baseline characteristics of the study population.

Total study population = 18,646	Control (9,875)	Intervention (8,771)
	n	%	n	%
**Sex (ratio = 0.98)**				
- Male	4,900	49.62	4,330	49.37
**Age groups:**				
- <10 y	2684	27.18	2342	26.7
- 10–19 y	2379	24.09	2240	25.54
- 20–29 y	1733	17.55	1688	19.25
- 30–39 y	1110	11.24	820	9.35
- 40–49 y	954	9.66	871	9.93
- >49 y	1015	10.28	810	9.23
**Ethnic groups:**				
- Ra-glai	8205	83.09	8233	93.87
- K'ho	1339	13.56	389	4.44
- Others (Kinh, Chu, Cham, Ede)	331	3.35	149	1.7
**Education level (age≥20, n = 9,001):**				
- None	2197	45.66	2033	48.53
- Primary school	2258	46.92	1843	44
- Secondary school or higher	341	7.09	313	7.47
- Missing	16	0.33		
**Occupation:**				
- None (children, students, retired people)	4376	44.31	3853	43.93
- Forest work (farming & other)	5242	53.08	4626	52.74
- Other (teacher, health staff…)	240	2.43	292	3.33
- Missing	17	0.17		
**Bed net use in the village:**				
- Sleep under ITN	8707	88.17	7381	84.15
- Sleep under an untreated bed net	494	5.0	816	9.3
- Missing	18	0.18	-	-
**Forest activities:**				
- Never	4176	42.29	3688	42.05
- Only during day	3244	32.85	3181	36.27
- Work and sleep in the forest	2438	24.69	1902	21.69
- Missing	17	0.17		
**Bednet/hammock° use in the forest (n = 4,340 forest workers):**				
- Sleep under ITN	1717	70.43	781	41.06
- Sleep in a hammock	319	13.08	230	12.09
- Sleep under ITN and in a hammock	80	3.28	555	29.18
- Sleep without bed-net and hammock	322	13.21	336	17.67
***Households (N = 3,652)***	***N = 1,866***		***N = 1,786***	
**House structure:**	**n**	**%**	**n**	**%**
- Thatched bamboo	950	50.91	644	36.06
- Wooden boards	374	20.04	632	35.39
- Dried mud	260	13.93	236	13.21
- Bricks	282	15.11	274	15.34
**Socio economic level:**				
- No radio, TV, motorbike	856	45.87	581	32.53
- Only a radio	517	27.71	643	36
- Only TV	99	5.31	105	5.88
- TV+radio (no motorbike)	94	5.04	174	9.74
- At least a motorbike (+/−radio, TV)	300	16.08	283	15.85
***Malariometric indices (Survey1, April 2004, N = 3,023)***	***N = 1,518***		***N = 1,505***	
Spleen rate, n%	29	1.9	6	0.4
Malaria infections (all species)	178	11.7	251	16.7
Asymptomatic infections (all species)	153	10.1	224	14.9

### Effect of intervention on malaria incidence

During the pre-intervention semester (July-December 2004), 647 new clinical malaria cases were identified by PCD, resulting in an incidence rate of 25.7/1000 person-semesters for the control and 41.9/1000 person-semesters for the intervention group (Figure 2.1), indicating an imbalance between the 2 study groups despite an earlier stratification on sero-prevalence. At the end of the 2-year follow up, malaria incidence had decreased in both groups (control group: 12.3 cases/1,000 person-semesters, IRR = 0.48; 95CI [0.28; 0.82]; intervention group: 9.7 cases/1,000 person-semesters, IRR = 0.23; 95CI [0.14; 0.38]) (Table 2). However, in 2006, the significant interaction term between the effect of time and intervention (semester1/2006: 0.45, 95CI [0.21; 0.98], p = 0.04; semester2/2006: 0.48, 95CI [0.25; 0.95], p = 0.03) indicated a 2-fold larger IRR in the intervention group (Table 2 & Figure 2.1), meaning that the malaria incidence in 2006 had decreased significantly more in the intervention than in the control group as compared to the baseline levels in 2004. The interaction terms did not change after adjustment of potential confounders such as age, bed net use, forest activity and wealth (data not shown). The difference in IRR between intervention and control groups already started in the last semester 2005 (interaction term = 0.66), though it did not reach statistical significance (p = 0.14). Similar results were observed when analysing *P. falciparum* and *P. vivax* incidence separately. For the former, the IRR in both trial groups as well as the interaction terms (semester1/2006: 0.44, 95CI [0.21; 0.95], p = 0.04; semester2/2006: 0.44, 95CI [0.21; 0.94], p = 0.04) were similar and equally significant to those observed for all malaria cases. For *P. vivax*, given the low number of cases detected, the interaction terms (semester1/2006: 0.59, 95CI [0.18; 1.94], p = 0.36; semester2/2006: 0.63, 95CI [0.24; 1.66], p = 0.33) did not reach statistical significance.

**Figure 2:**
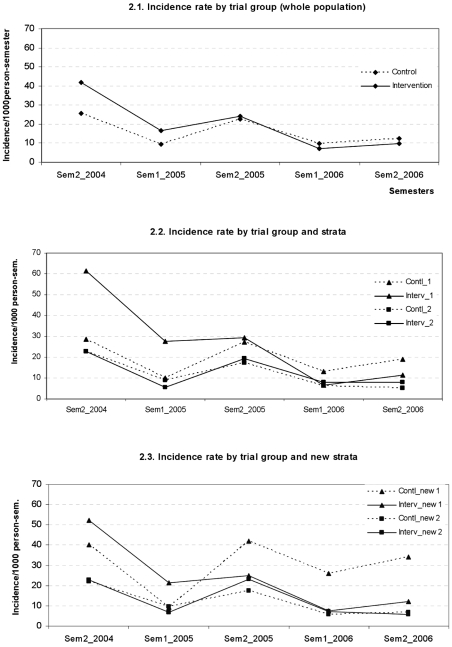
Evolution of malaria incidence rates by semester and trial groups. 2.1 Incidence rate by trial group (whole population); 2.2 Incidence rate by trial group and strata; 2.3 Incidence rate by trial group and new strata.

**Table 2:** Multivariate analysis for the risk of clinical malaria using survey-Poisson regression with the interaction between survey and trial group.

2.1. Effect of time on malaria incidence					
Control group
Semester	New cases/person-sem	Incidence rate*	IRR	[95% CI]	P-value
Semester 2/2004	264/10,262.2	25.73	1		
Semester 1/2005	99/10,403.1	9.52	0.37	[0.23; 0.60]	<0.001
Semester 2/2005	237/10,561.1	22.44	0.87	[0.55; 1.38]	0.54
Semester 1/2006	105/10,640.1	9.87	0.38	[0.21; 0.69]	0.003
Semester 2/2006	132/10,706.5	12.33	0.48	[0.28; 0.82]	0.011
**Intervention group**					
**Semester**	**New cases**	**Incidence rate** *	**IRR**	**[95% CI]**	**P-value**
Semester 2/2004	383/9132.4	41.94	1		
Semester 1/2005	151/9212.4	16.39	0.39	[0.19; 0.82]	0.016
Semester 2/2005	226/9317.0	24.26	0.58	[0.41; 0.82]	0.004
Semester 1/2006	68/9386.7	7.24	0.17	[0.1; 0.32]	<0.001
Semester 2/2006	92/9453.5	9.73	0.23	[0.14; 0.38]	<0.001

2.2. Interaction term between time and study group			
Whole population
Semester	Interaction term#	[95% CI]	P-value
Semester 1/2005	1.06	[0.44; 2.53]	0.90
Semester 2/2005	0.66	[0.38; 1.16]	0.14
Semester 1/2006	**0.45**	[0.21; 0.98]	0.046
Semester 2/2006	**0.48**	[0.25; 0.95]	0.037
**High endemicity stratum (stratum1)**			
Semester 1/2005	1.29	[0.36; 4.59]	0.65
Semester 2/2005	0.50	[0.37; 0.66]	<0.001
Semester 1/2006	**0.23**	[0.08; 0.65]	0.001
Semester 2/2006	**0.28**	[0.13; 0.60]	0.005
**“New stratum 1”** *			
Semester 1/2005	1.81	[0.58; 5.63]	0.26
Semester 2/2005	0.46	[0.34; 0.61]	<0.001
Semester 1/2006	**0.22**	[0.09; 0.51]	0.003
Semester 2/2006	**0.27**	[0.14; 0.52]	0.002

* Incidence rate = new cases/1,000person-semester.

* “New stratum 1” = in a sensitivity analysis, clusters were re-assigned to 2 new strata based on the December 2004 parasite rate (New stratum 1≥20%; New stratum2<20%).

# Point estimate of the interaction term obtained after exponentiation.

The greater effect on malaria incidence observed in the intervention group occurred mainly in the high endemicity stratum (“stratum 1”), while no difference between study groups was observed in the low endemicity stratum (“stratum 2”; Figure 2.2). This was confirmed in the regression model with a significant interaction between the effect of time and intervention in stratum 1, while no additional effect of intervention could be found in stratum 2 (Table 2). The 2-fold larger IRR in the intervention group was already significant in 2005 (semester2/05, interaction term = 0.50; 95%CI [0.37; 0.66], p<0.001) and the difference between groups continued to increase in 2006 (semester2/06, interaction term = 0.28; 95%CI [0.13; 0.60], p = 0.005), despite the absence of a malaria peak in the 2^nd^ semester of 2006 (Figure 2.2). In the low endemicity stratum, the IRR was similar in both groups (control: IRR = 0.23, 95%CI [0.09; 0.58]; intervention: IRR = 0.35, 95%CI [0.19; 0.63]).

The analysis carried out with the new strata confirmed that the stronger effect in the intervention group was not due to its higher starting incidence rate at the beginning of the intervention. In the new high incidence stratum, pre-intervention incidence rates were more comparable between control (40/1000person-semesters) and intervention (52/1000person-semesters) groups. Malaria incidence rapidly decreased in the intervention group, to 12/1000 person-semesters in the second half of 2006 (IRR = 0.23, 95%CI [0.12; 0.45]), while it remained almost unchanged in the control group (from 40 to 34/1000person-semesters, IRR = 0.85, 95%CI[0.82; 0.90]) (Figure 2.3). The interaction terms between time and intervention were similar to those observed in the original stratum 1, confirming the independent effect of the intervention (Table 2).

### Effect of intervention on malaria prevalence

Before the distribution of LLIH, in December 2004, the overall parasite rate was 17.8% (727/4090), with the tendency of a higher prevalence in the intervention (22.1%, 446/2022) than in the control group (p = 0.12) (Figure 3.1). As for the incidence, the evolution of malaria prevalence across the 5 consecutive surveys was measured within each study group and then compared between groups using a survey logistic regression model in which an interaction term between time and intervention was fitted (Table 3). The effect of potential confounders such as age, bed net use, forest activity and wealth were assessed, and as the interaction terms did not change, the simplest model was chosen. After 1 year, in the intervention group, the odds of malaria infection as compared to baseline had decreased by 65% (OR = 0.35; 95CI [0.29; 0.43]) and by 85% after 2 years (OR = 0.15; 95CI [0.09; 0.26]) (both p<0.001)(Table 3). In the control group, the reduction was by 43% (OR = 0.57; 95CI [0.48; 0.69]) and by 74% (OR = 0.26; 95CI [0.20; 0.35], both p<0.001) respectively after one and two years (Table 3). The difference between intervention and control groups was at the highest during the last semester of 2005 and the first of 2006 when the interaction terms were 0.62 (95%CI [0.48; 0.79]) and 0.63 (95%CI [0.42; 0.96]), respectively, indicating a 1.6-fold greater reduction in the intervention than in the control group. As malaria prevalence at the last survey was extremely low in both groups, no significant difference could be observed. When considering *P. falciparum* and *P. vivax* separately, the trend was similar to the overall prevalence though the interaction term was not statistically significant anymore because of the lower power (data not shown).

**Figure 3:**
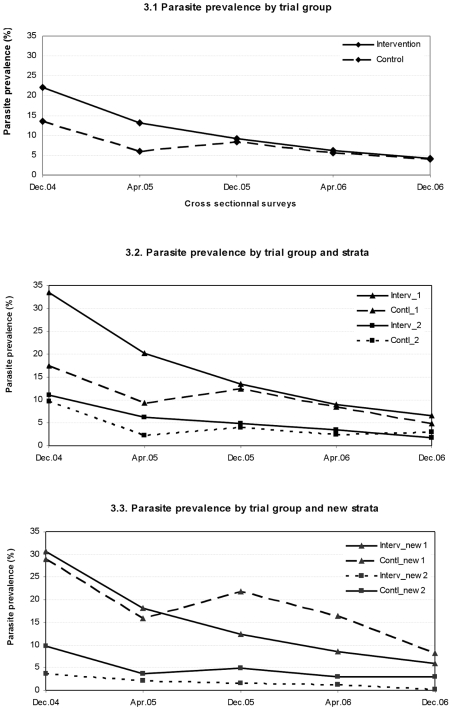
Evolution of malaria prevalence across five consecutive cross-sectional surveys. 3.1 Parasite prevalence by trial group (whole population); 3.2 Parasite prevalence by trial group and strata; 3.3 Parasite prevalence by trial group and new strata.

**Table 3:** Multivariate analysis for the risk of malaria infection using survey logistic regression with the interaction between survey and trial group.

3.1 Effect of time on the risk of malaria infection					
Control group
Survey	n/N	Prevalence (%)	OR	[95% CI]	P-value
2/2004	281/2,068	13.59	1		
1/2005	126/2,081	6.05	0.41	[0.33; 0.52]	<0.001
2/2005	173/2,089	8.28	0.57	[0.48; 0.69]	<0.001
1/2006	116/2,102	5.52	0.37	[0.25; 0.56]	<0.001
2/2006	80/2,018	3.96	0.26	[0.20; 0.35]	<0.001
**Intervention group**					
**Survey**	**n/N**	**Prevalence**	**OR**	**[95% CI]**	**P-value**
2/2004	446/2,022	22.06	1		
1/2005	270/2,061	13.10	0.53	[0.40; 0.72]°	<0.001
2/2005	183/2,014	9.09	0.35	[0.29; 0.43]°	<0.001
1/2006	131/2,095	6.25	0.24	[0.20; 0.28]°	<0.001
2/2006	84/2,045	4.11	0.15	[0.09; 0.26]°	<0.001

3.2 Interaction term between time and trial group°			
Whole population
Survey	Interaction term#	[95% CI]	P-value
2/2004	1		
1/2005	1.30	[0.90; 1.87]	0.15
2/2005	0.62	[0.48; 0.79]	0.013
1/2006	0.63	[0.42; 0.96]	0.037
2/2006	0.58	[0.31; 1.08]	0.137
**High endemicity stratum (stratum 1)**			
2/2004	1		
1/2005	1.02	[0.64; 1.63]	0.91
2/2005	0.46	[0.34; 0.61]	<0.001
1/2006	0.44	[0.27; 0.73]	0.005
2/2006	0.57	[0.25; 1.28]	0.15
**New stratum 1** *			
2/2004	1		
1/2005	1.08	[0.73; 1.58]	0.67
2/2005	0.46	[0.37; 0.58]	<0.001
1/2006	0.44	[0.23; 0.83]	0.018
2/2006	0.64	[0.34; 1.22]	0.15

°Representing the ratio between intervention and control group for the effect of time (odds time t/odds time 0).

* “New stratum 1” = in a sensitivity analysis, clusters were re-assigned to 2 new strata based on the December 2004 parasite rate (New stratum 1≥20%; New stratum2<20%).

# Point estimate of the interaction term obtained after exponentiation.

Similarly to the incidence, the stronger effect on malaria prevalence in the intervention group was mainly observed in the high endemicity stratum, i.e. intervention from 33.5% in Dec.04 to 6.6% in Dec.06; control from 17.4% to 4.9%. In the low endemicity stratum, the prevalence in both study groups decreased similarly, i.e. from 11.1% to 1.7% in the intervention and from 9.6% to 3.0% in the control group (Figure 3.2). The significant interaction terms for the 3^rd^ (0.46, 95CI [0.34; 0.61], p<0.001) and 4^th^ surveys (0.44, 95CI [0.27; 0.73], p = 0.005) found in the multivariate survey logistic regression model in the high endemicity stratum, confirmed that the observed effect on prevalence was 2-fold larger in the intervention as compared to the control group (Table 3), one and one and half years after the introduction of LLHI. No significant difference in reductions between groups was observed in the low endemicity stratum (Figure 3.2). When re-stratifying the clusters according to the prevalence at the December 2004 survey, results similar to those in the original high endemicity stratum were obtained (Figure 3.3).

The number of malaria cases averted by the intervention was estimated at 10.5 per 1,000 persons for the last semester of 2006 compared to the last semester 2004. This estimation is higher and occurred earlier for the high endemicity stratum, i.e. 29 cases per 1,000 individuals, both in the second semesters of 2005 and 2006. Such estimations refer to the period of high transmission, i.e. between July and December. Similarly, the number of malaria infections averted after 1 year of intervention was 5.6/100 individuals in the whole population and 15.7/100 individuals in the high endemicity stratum.

### LLIH use

According to data collected during the December surveys in 2005 and 2006, LLIH use in the intervention group's villages was extremely high, around 93% (1890/2016 and 1898/2045), and was lower among people sleeping in the forest, 86% (283/329) and 83% (232/280), respectively. LLIH use during the evening or at night in the villages increased from 35% (671/1890) in 2005 to 60% (1146/1898) in 2006, while among workers staying in the forest overnight it remained low, 33.4% (110/329) in 2005 and 23.6% (66/280) in 2006.

## Discussion

Both malaria incidence and prevalence decreased significantly faster in the clusters where LLIH had been distributed, indicating that this intervention had a beneficial effect on the malaria burden in these remote Vietnamese villages. This occurred despite the relatively low LLIH use at the time the vector was active, i.e. during the evening and at night time, and an even lower use among workers staying in the forest overnight, a practice strongly associated to the risk of malaria infection, with a previously estimated population-attributable fraction of 53% [7]. Indeed, the reason for evaluating the effectiveness of a new control tool such as the LLIH originated from the observation done in a neighbouring province that, despite a high ITN coverage, malaria remained a problem, with a risk for *P. falciparum* clinical malaria almost 4-fold higher in people regularly working but not sleeping in the forest and 8-fold higher in those sleeping in the forest but using a bed net at home [7]. The low risk of malaria infection in children <9 years old, the youngest malaria infected person was a 7 years old child (Erhart, personal communication), indicated that in this area malaria was essentially an occupational disease, with adults having between a 3- to 9-fold higher risk of malaria infection compared to the 0–19 years old [7]. This situation was mostly explained by the vector *Anopheles dirus*, an extremely efficient species for malaria transmission, present in the SEA forest zones and characterized by early biting, exophagy and exophily. The first two behaviours make it less vulnerable to ITNs and the latter to IRS [6], [15]. Therefore, for controlling malaria in this specific situation there was the need of devising a tool such as the LLIH, able to protect people, more specifically forest workers, exposed to *A. dirus* infectious bites. The impact observed in the present study is probably higher than the one that would have been predicted by considering the LLIH timing of use and the coverage, particularly for the high risk groups. This is probably due to the several differences between the provinces of Binh Thuan, where previous studies on malaria epidemiology were carried out [7], and Ninh Thuan, where the LLIH study was done. In the latter, the villages were surrounded by the forest and malaria transmission, supported by *A. dirus*, may have occurred in the village itself. The higher prevalence and incidence of clinical attacks in young children (<10) as compared the older age groups, unlike Binh Thuan, is consistent with this hypothesis [12]. Therefore, even if LLIH use among forest workers was not extremely high, the coverage obtained in the villages was sufficient to prevent a substantial number of clinical cases and malaria infections. It is worth noticing that the effect of the intervention mainly occurred in the villages belonging to the high endemicity stratum while in the low endemicity stratum no additional effect of the LLIH to the already significant decrease experienced in the control group during the study period was observed.

In 2003, a large malariometric survey aiming at identifying the villages with the highest prevalence of malaria infection was carried out as a preparation to the LLIH study [8]. Both the prevalence of malaria infection and that of malaria antibodies showed large variations between villages, reflecting the clustering of malaria transmission in space and time. Clusters were stratified according to sero-prevalence, considered to best reflect the malaria infection risk in the 6 months prior the survey. Unfortunately, despite the stratification, important difference remained, with the intervention group having a much higher prevalence and incidence than the control group. Therefore, the analysis had to take into account such imbalance and this was the main reason why the effect of the intervention was estimated with an indirect rather than a direct approach, i.e. by comparing between groups, the rate of decrease over the 2-year follow-up period. Another reason for adopting such an approach was that malaria morbidity decreased substantially in the control group as well, so that at the end of the observation period both the incidence of clinical cases and the prevalence of infection were extremely similar between the 2 study groups. A direct comparison would have overlooked the actual effect of the LLIHs, i.e. a significantly faster decrease in the intervention clusters, mainly in the high endemicity stratum.

The observed reduction in the malaria incidence and prevalence in the control group in both strata may be explained by the presence of VHWs who were able to promptly diagnose clinical malaria with RDT and provide adequate treatment at village level [12]. Such system of community-based monitoring was necessary for the identification of clinical cases so that the effect of LLIHs could be measured. However, it also played an important role in decreasing the malaria burden in these communities [12], though it is unclear whether this would continue to decrease after reaching the pre-elimination level of a slide positivity rate lower than 5% [1].

Though at the end of the study malaria prevalence and incidence had dramatically decreased in both groups, such reduction was much stronger in the intervention group, as confirmed by the significant interaction between the effect of time and intervention in the multivariate regression models. The effect of LLIH on malaria prevalence could already be observed at the end of 2005, with a 1.6-fold greater effect until reaching similar prevalence to the one in the control group, around 9%, and then evolving similarly. Conversely, the significant effect of LLIH on malaria incidence could be observed only during the second year of the intervention, though in the high endemicity stratum such an effect was already evident in the first year post intervention and continued during the second year to attain a 3.6-fold larger effect as compared to the control group. The robustness of our estimations is supported by the similar results obtained when re-analyzing data with the new stratification that made the study groups comparable in terms of pre- intervention morbitity. The stronger impact of LLIH in clusters with the highest malaria burden is confirmed by the larger number of clinical cases averted in the high endemicity stratum during the second year of the intervention, i.e. almost 30/1,000 person-semester as compared to 10.5/1,000 person-semester over the whole intervention group.

Insecticide-treated materials, such as permethrin-impregnated bedsheets [16] or LLIH, could be used in places where standard methods such as ITN or IRS may not be appropriate or have little impact. LLIH appear to be a practical control tool in remote places, specifically where forest malaria is still a problem. Though both LLIH and the community-based monitoring system based on VHW eventually reached after 2 years the same results, i.e. similar prevalence and incidence of malaria, the complexity of setting up and maintaining the latter should not be underestimated, while LLIH can be distributed at once with no need of continuous supervision for ensuring the quality of the diagnosis and treatment. In addition, considering the high coverage obtained in this study within a relatively short period, LLIH may be rapidly accepted in ethnic minorities living in remote and forested areas.

This is the first large community-based study on the effectiveness of LLIH in controlling forest malaria. The only field testing of LLIH by using volunteers sleeping in concrete block experimental huts was carried out in Benin and showed that LLIH provided protection similar to mosquito coils against endophagic mosquitoes [17]. However, LLIH were more cost-effective and user-friendly than mosquito coils as these needed to be replaced every night. The protection against exophagic mosquitoes has not been established yet, but the results of this trial indicate that it may be substantial. Further field trials with other WHOPES-recommended long lasting insecticidal nets and possibly with a better hammock design (size, material) and with a higher coverage are needed to confirm these first encouraging results.

In conclusion, as the targets of the newly-launched Global Malaria Action Plan include the 75% reduction of the global malaria cases by 2015 as compared to the 2000 levels and eventually the elimination/eradication of malaria in the long term, LLIH may represent an additional tool for reaching such objectives, particularly in areas where standard control tools have a modest impact, such as in remote and forested areas of Southeast Asia and possibly South America.

## Supporting Information

Checklist S1 CONSORT Checklist
(0.35 MB DOC)

Protocol S1 Trial Protocol
(0.11 MB DOC)
