# Supplementary material for: Long-Lasting Insecticidal Hammocks for Controlling Forest Malaria: A Community-Based Trial in a Rural Area of Central Vietnam
Source: PLoS One. 2009 Oct 7;4(10):e7369. doi: 10.1371/journal.pone.0007369 (PMC2752990; doi:10.1371/journal.pone.0007369)
Supplement: Checklist S1 — CONSORT Checklist (0.35 MB DOC) [file pone.0007369.s001.doc]

**Long-lasting insecticidal hammocks for controlling forest malaria: a community-based trial in a rural area of Central Vietnam**

Ngo Duc Thang, Erhart Annette, Niko Speybroeck, Nguyen Xuan Xa, Nguyen Ngoc Thanh,

Pham Van Ky, Le Xuan Hung, Le Khanh Thuan, Coosemans Marc, D’Alessandro Umberto

**Based on:**

Campbell MK, Elbourne DR, Altman DG. Consort statement: extension to cluster randomized trials. BMJ 2004 Mar 20; 328: 702-708

**Item numbers:**

**Title and abstract (1)**: **How participants were allocated to interventions (eg, “random allocation,” “randomized,” or “randomly assigned”), *specifying that allocation was based on clusters.*** The title mentions that this is community-based trial, implying the allocation by cluster. In the abstract, in the Methods and findings section it is stated: “Thirty villages (population 18,646) were assembled in 20 clusters (1,000 individuals per cluster) that were randomly allocated to either the intervention or control group after stratification according to the pre-intervention *P. falciparum* antibody prevalence (<30%; ≥30%).”

**Introduction: Background (2): Scientific background and explanation of rationale, *including the rationale for using a cluster design.*** This is addressed in the introduction section where the rationale for this study is explained. As this was an effectiveness study, the only possible design was a cluster one.

**Methods: Participants (3)**: **Eligibility criteria for participants *and clusters and the settings and locations where the data were collected***. This is addressed in the methods in the sections ***Study site & population*** and ***Sample size calculation and randomization process.*** All residents in the study villages willing (on a voluntary basis) to participate in the study (information meetings were held with community leaders and representatives during the baseline period (from April 2004 to December 2004)) were included in the study population. Nobody refused to take part in the study, rather the population was very eager to participate, possibly because of the remoteness of the study area. The study population was represented by all individuals living in the area throughout the study, until its completion, i.e. new comers and newborns were included throughout the study.

**Interventions (4): Precise details of the interventions intended for each group, *whether they pertain to the individual level, the cluster level, or both, and how and when they were actually administered****.* This is addressed in the **Methods** section under the sub-heading ***Intervention: Long Lasting Insecticidal Hammocks (LLIH)*** where the details of the intervention are revealed. 7,000 LLIH were distributed to all individuals aged 10 years old and above.

**Objectives (5): Specific objectives and hypotheses and whether they pertain to the individual level, the cluster level, or both.** Due to space limitations, the draft manuscript does not include the specific objectives and hypotheses. Essentially, the overall objective was to evaluate the effectiveness of LLIH as a new strategy for malaria control in forested areas of Central Vietnam. The specific objectives were the following:

- To characterise malaria epidemiology and dynamics of transmission in the forested areas of Central Vietnam;
- To measure the effectiveness of LLIH in preventing malaria infections among forest workers and other villagers;
- To establish the cost-effectiveness of LLIH and their impact on the local economy.

**Outcomes (6): Report clearly defined primary and secondary outcome measures, *whether they pertain to the individual level, the cluster level, or both, and when applicable, any methods used to enhance the quality of measurements (eg multiple observations, training of assessors.*** The case definition is in the Methods section. Primary outcomes were the incidence of clinical malaria cases (measured by passive case detection) and the prevalence of malaria infection (measured by bi-annual cross sectional surveys).

**Sample size (7): How *total* sample size was determined *(including method of calculation, number of clusters, cluster size, a coefficient of intracluster correlation (ICC or k, and an indication of its uncertainty),* and, when applicable, explanation of any interim analyses and stopping rules.**

This is addressed in the section “***Sample size calculation and randomization process”***.

The sample size was estimated taking into account the cluster randomized design and assuming a reduction of 30% in malaria prevalence and 33% in malaria incidence (at 5% significance level and 80% power) within 2 years in the intervention as compared to the control group. Ten clusters (1,000 inhabitants each) per study group were necessary, and, within each cluster, a cohort of 160 individuals randomly selected for the bi-annual surveys were included in the study. In each cluster, 60 children 2-9 years old were later added to the cohort following the high prevalence of malaria infection in this age group at the baseline survey (April 2004). Each cluster comprised one to three neighbouring villages, according to their size, to total about 1,000 individuals. The coefficient of between cluster variation k (following Hays & Bennett, 1999) used for the sample size estimation was k=0.3

**Randomization: Sequence generation (8): Method used to generate the random allocation sequence, including details of any restriction (eg, blocking, stratification, *matching*)**.

This is addressed in the section “***Sample size calculation and randomization process”***.

Before randomisation, clusters (numbered from 01 to 20) were stratified according to the prevalence of antimalarial antibodies determined in a 2003 survey that showed high variability between villages, with values ranging from 0 to 75%. A “high endemicity” (*P.falciparum* sero-prevalence≥30%) and “low endemicity”(sero-prevalence<30%) strata were defined, resulting in balanced groups within each stratum, i.e. *P.f* seroprevalence of 35.2% (95% CI: 24.9; 47.2) in the control *versus* 42.0% (95% CI 26.0; 60.3) in the intervention group in the high endemicity stratum, and 20.0% (95% CI: 15.1; 26.0) *vs* 19.9% (95% CI: 15.2; 25.5) in the low endemicity stratum. A randomisation list was produced for each stratum.

**Allocation concealment (9): Method used to implement the random allocation sequence *specifying that allocation was based on clusters rather than individuals and* clarifying whether the sequence was concealed until interventions were assigned**.

The randomisation was done beforehand on the numbered clusters, without knowing the name of the villages involved. Therefore, the actual allocation of a specific village (belonging to a given cluster) to the intervention and control group was not known until the implementation of the intervention.

**Implementation (10): Who generated the allocation sequence, who enrolled participants, and who assigned participants to their groups**.

See above.

**Blinding (masking) (11): Whether participants, those administering the interventions, and those assessing the outcomes were blinded to group assignment. If done, how the success of blinding was evaluated**.

The intervention could obviously not be blinded since only the intervention group received LLIH. Reading of blood slide was done blind to the origin of the slide, i.e. the reader did not know whether it was from the intervention or control group. The data base was cleaned and locked without knowing the group allocation.

**Statistical methods (12): Statistical methods used to compare groups for primary outcome(s), *indicating how clustering was taken into account,* methods for additional analyses, such as subgroup analyses and adjusted analyses**.

Clustering was taken into account in all statistical analysis by using the svy command in Stata (svylogit for the analysis of prevalence data, and svypois for the analysis of incidence data) taking into account the study design (primary sampling unit=cluster; strata=stratum 1 or 2).

**Results: Participant flow (13): Flow of *clusters and* individual participants through each stage (a diagram is strongly recommended). Specifically, for each group report the numbers of *clusters and* participants randomly assigned, receiving intended treatment, completing the study protocol, and analyzed for the primary outcome. Describe protocol deviations from trial as planned, together with reasons**.

In Table 2 the evolution of the study population in each group is specified (person-semesters). In table 3, the number of survey participants is detailed for each group. We can provide additional information if required.

**Recruitment (14): Dates defining the periods of recruitment and follow-up**.

The dates of identification of the cluster, of the pre-intervention activities and of the follow up of the intervention are clearly mentioned in the results section. See also Table 2 and 3 for the semesters.

**Baseline data (15): Baseline information for each group *for the individual and cluster levels a*s applicable**.

See table 1.

**Numbers analyzed (16) Number *of clusters* and participants (denominator) in each group included in each analysis and whether the analysis was by intention-to-treat. State the results in absolute numbers when feasible (eg, 10/20, not 50%)**.

The analysis was intention-to-treat. The number of cluster is reported as well as the number of individuals in the population and in the surveys.

**Outcomes and estimation (17): For each primary and secondary outcome, a summary of results for each group *for the individual or cluster level* *as applicable* , and the estimated effect size and its precision (eg, 95% confidence interval) *and a coefficient of intra cluster correlation (ICC or k) for each primary outcome.***

Table 2 and 3 and the Figures report the summary results as well as the 95% CI. .

**Ancillary analyses (18): Address multiplicity by reporting any other analyses performed, including subgroup analyses and adjusted analyses, indicating those pre-specified and those exploratory**.

No ancillary analysis has been done. However, in an attempt to make the 2 groups comparable, analysis on a different stratification has been done. Both the analyses on the original and new stratification are reported.

**Adverse events (19): All important adverse events or side effects in each intervention group**.

No adverse event reported.

**Discussion: Interpretation (20): Interpretation of the results, taking into account study hypothesis, sources of potential bias or imprecision and the dangers associated with multiplicity of analyses and outcomes**. This has extensively been addressed in the discussion.

**Generalisability (21): Generalisability (external validity) *to individuals and/or clusters (as relevant)* of the trial findings**. This has also been addressed in the discussion.

**Overall evidence (22): General interpretation of the results in the context of current evidence**. This is the first large study on the effectiveness of LLIH in South East Asia and in the world. The applicability of the results is discussed.

1. Piaggio G, Elbourne DR, Altman DG, Pocock SJ, Evans SJ. Reporting of noninferiority and equivalence randomized trials: an extension of the CONSORT statement. JAMA. 2006 Mar 8; **295**(10):1152-60.
