# Supplementary material for: Long-Lasting Insecticidal Hammocks for Controlling Forest Malaria: A Community-Based Trial in a Rural Area of Central Vietnam
Source: PLoS One. 2009 Oct 7;4(10):e7369. doi: 10.1371/journal.pone.0007369 (PMC2752990; doi:10.1371/journal.pone.0007369)
Supplement: Protocol S1 — Trial Protocol (0.11 MB DOC) [file pone.0007369.s002.doc]

# PROJECT PROPOSAL:

# *EFFECTIVENESS OF INSECTICIDE-TREATED HAMMOCKS FOR CONTROLLING FOREST MALARIA IN VIETNAM*;

# ------------

# 1. PARTNER/ ORGANIZATION MAKING THE APPLICATION:

Prince Leopold Institute of Tropical Medicine (ITM), Nationalestraat 155,B-2000 Antwerp/ BELGIUM. The ITM is an international research and training institution supported by public and private funds. It is managed by the Board of Governors whose members are from Belgian universities, federal and regional institutions.

| ***- Principal investigator:*** | ***Study co-ordinators:*** |
| --- | --- |
| Pr. Umberto D’Alessandro, *MD, Msc, PhD* Head of the Unit of Epidemiology  Department of Parasitology | Dr. Annette ERHART, *MD, Msc*  Research assistant- Unit of Epidemiology  Pr. Marc Coosemans, Msc, PhD  Head of the unit of Entomology  Dept of Parasitology |

# PROJECT DESCRIPTION

## GENERAL SITUATION

In Vietnam, over the last ten years, malaria morbidity and mortality have been substantially reduced as a result of an effective national control program (1;2). However, malaria cannot be considered “under control” since the threat of resurgence still exists. Malaria remains endemic in the central highlands, home of ethnic minorities, because of the complex epidemiology of forest malaria. These mountainous and forested areas attract also many migrants from non-endemic provinces and from areas bordering Laos and Cambodia (3;4). Such population movements and specific ecological factors contribute to maintain efficiently malaria transmission and to the re-introduction of malaria parasites where they had disappeared. A recent community-based study in Central Vietnam showed that regular forest activity was a very strong risk factor for malaria infection (5). Untreated bed nets had a significant protective effect for villagers, except for those regularly sleeping in the forest, who suffered a significantly higher number of clinical attacks. This has major economic implications for rural communities as clinical malaria means a substantial number of working days lost and a reduction of the income. Therefore, there is the need of targeting this high-risk group with new interventions based on insecticide-treated material to be tested in field trials (6;7). Hammocks are extensively used by people working in the forest and treating them with insecticide could achieve a good individual protection. In Cambodia, where forest malaria is also common, insecticide-treated hammock nets are used although their impact on malaria morbidity and on the local economy has never been quantified (8). We propose to carry out an extensive evaluation of insecticide-treated hammocks (ITH) targeted to forest workers in order to establish their usefulness for controlling malaria in South-East Asia.

## EXTRA

This is a new project that originates by research carried out in collaboration with Vietnamese colleagues at the National Institute of Malariology, Parasitology and Entomology (NIMPE) in Hanoi, Vietnam, with whom we have established collaboration since 1992 (9-24). Although malaria is being successfully controlled in Vietnam, active foci, mainly in forested areas, continue to maintain transmission. Therefore, the search for an appropriate intervention to deal with this problem is timely and needed.

## PROJECT TITLE: Effectiveness of insecticide-treated hammocks for controlling forest malaria: a community-based trial.

## PROJECT DESCRIPTION

***Overall objective:*** To evaluate the effectiveness of insecticide-treated hammocks (ITH) as a new strategy for malaria control in forested areas of Central Vietnam.

***Specific objectives:***

- To characterise malaria epidemiology and dynamics of transmission in the forested areas of Central Vietnam;
- To measure the effectiveness of ITH in preventing malaria infections among forest workers and other villagers;
- To establish the cost-effectiveness of ITH and their impact on the local economy.
- To increase research capabilities by training a young Vietnamese scientist at PhD level.

***Milestones:*** *DECEMBER 2003*: complete census of study villages, updated yearly for the following 2 years; *FEBRUARY 2004*: Passive case detection of malaria cases set up and running until December 2006; *JUNE 2004*: Health economic study started, first results available by December 2006; *DECEMBER 2004*: Intervention (ITH) is implemented in half of the study villages; *JUNE 2005*: Dynamics of malaria transmission known; *DECEMBER 2006*: 7 malariometric surveys (2 per year) completed and results available; Insecticide-treated hammock distributed to control villages if a significant difference in the intervention villages is found; *JUNE 2007*: publication first results and information to health authorities; *DECEMBER 2007*: completion PhD by a Vietnamese researcher.

***Planned methodology****:*Community-based, cluster-randomised trial in which half of the study villages will received ITH after a first year of baseline data collection. Twenty villages of about 1000 inhabitants situated in a forested area in Binh Thuan province will be identified. Sample size calculated on the basis that the cluster unit is the village and that the intervention will reduce malaria morbidity (malaria antibodies, clinical attacks) in intervention villages by at least 30% (5% level and 90% power). This takes into account the (estimated) coefficient of variation of true proportions between communities and the cluster effect. **Baseline year**: complete census of the study population to be used to randomly select, for each village, a cohort of 50 regular forest workers and of 150 villagers. A Knowledge, Attitudes and Practices study (forest activities, hammock use and basic economic indicators) and 2 malariometric surveys (one at the beginning and one at the end of the transmission season) to collect baseline information and allow stratification of the villages according to key variables (for example malaria prevalence and seropositivity, acces to treatment, etc.) and their randomisation into 2 groups within each stratum. Passive detection of clinical cases (collection of blood slides and treatment) set up and running between surveys. Quarterly entomological collections (human landing catches) within and around 2 study villages for species identification and Entomological Inoculation Rate calculation (EIR). **Intervention** (2 years): ITH distributed to all adults (age>15 years) in the 10 intervention villages. Bi-annual malariometric surveys and passive detection of clinical cases continued in all villages. Evolution of malaria antibodies prevalence and incidence of clinical malaria among the cohorts under surveillance determined and compared between intervention and control villages. Direct and indirect costs linked to malaria clinical attacks will be determined and the cost involved in preventing a clinical case will be calculated.

## PROJECT OPERATIONS

**Start**: November 2003, **Phases/stages**: One year baseline data collection; randomisation villages in 2 groups and distribution of ITH in the intervention villages at the end of the baseline year; Insecticide re-treatment of ITH every year for 2 years; Continued surveillance malaria morbidity for 2 years; Health economic study during the intervention years; Data analysis and result publications; discussion with Vietnamese authorities on scaling up if intervention successful. **Measures**: Coverage and compliance of ITH in study villages; incidence of infection (malaria antibodies) and clinical attacks (passive surveillance) and comparison between intervention and control villages; direct and indirect costs linked to clinical malaria; cost of preventing a clinical attack with ITH; EIR, vector species and their behaviour. **Deadlines**: see Milestones. **End**: Field activities ended by December 2006; Data analysis and reports/publications by June 2007; PhD completion by December 2007. **Implementation**: ITM is responsible for the overall co-ordination and management of the project; NIMPE, Hanoi, is responsible for the local implementation and management of the project; the provincial malaria station in Binh Thuan is responsible for the implementation of the ITH at village level and for the local co-ordination and supervision of field activities.

## LEVERAGE

# The project will improve our knowledge on the dynamics of malaria transmission in forested area and consequently key determinants to be targeted by control activities will be identified.

- The project will evaluate a new intervention for preventing forest malaria, a major problem for south-eastern Asian countries, and, if successful, it could change the approach to malaria control in this region.
- Large-scale implementation of ITH could increase the quality of life of people living in remote places, usually local minorities, by reducing the burden of malaria and consequently improving the local economy.
- The project will further consolidate the scientific collaboration between ITM and NIMPE whose competitiveness for international grants will increase.

## 2.7 INCLUSION OF AND DELEGATION OF TASKS: see section 2.5, Implementation.

## 2.8 EXIT OR HAND OVER SCENARIO: This project will be included into the long-term institutional collaboration between ITM and NIMPE for which an agreement exists until 2007. Such collaboration is funded by the Belgian co-operation and it is usually renewed every 5 years. Therefore, this proposal fits perfectly into this framework, as the collaboration between the 2 institutions will continue even after the completion of the proposed study.

# 2.9 COMPETITION: To our knowledge this type of research question has never been explored in Vietnam and no other research groups are currently working on it.

# PROJECT MANAGEMENT

- ***Scientific management*:** Prof. D’Alessandro and Dr Erhart will co-ordinate all aspects of the project in collaboration with colleagues at NIMPE. Communication and discussion of the results will be done at yearly meeting. The co-ordination will be assisted by a local full time project assistant taking care for the day-to-day activities in Vietnam.

### *Administrative management:* ITM-Antwerp has a long practice in administration of externally funded projects. General management will be performed by a professional administrator of ITM-Antwerp, who will interact with administrative responsible of NIMPE and provide administrative reports to the co-ordinator.

- ***Evaluation:*** The institutional collaboration project between ITM and NIMPE has been recently evaluated by external auditors. The project was evaluated extremely well (see joint document)

**TIMETABLE**

| **ACTIVITIES** | **YEAR 1** | | | | YEAR 2 | | | | **YEAR 3** | | | | **YEAR 4** | | | |
| --- | --- | --- | --- | --- | --- | --- | --- | --- | --- | --- | --- | --- | --- | --- | --- | --- |
| **Quarters** | **1** | **2** | **3** | **4** | **1** | **2** | **3** | **4** | **1** | **2** | **3** | **4** | **1** | **2** | **3** | **4** |
| Preparation, Training |  |  |  |  |  |  |  |  |  |  |  |  |  |  |  |  |
| Census |  |  |  |  |  |  |  |  |  |  |  |  |  |  |  |  |
| KAP survey |  |  |  |  |  |  |  |  |  |  |  |  |  |  |  |  |
| Entomological surveys |  |  |  |  |  |  |  |  |  |  |  |  |  |  |  |  |
| Health economic study |  |  |  |  |  |  |  |  |  |  |  |  |  |  |  |  |
| Intervention (ITH) |  |  |  |  |  |  |  |  |  |  |  |  |  |  |  |  |
| Cross-sectional surveys |  |  |  |  |  |  |  |  |  |  |  |  |  |  |  |  |
| Passive case detection |  |  |  |  |  |  |  |  |  |  |  |  |  |  |  |  |
| Laboratory work |  |  |  |  |  |  |  |  |  |  |  |  |  |  |  |  |
| Supervision |  |  |  |  |  |  |  |  |  |  |  |  |  |  |  |  |
| Data entry, cleaning |  |  |  |  |  |  |  |  |  |  |  |  |  |  |  |  |
| Data analysis, |  |  |  |  |  |  |  |  |  |  |  |  |  |  |  |  |
| Discussion results, |  |  |  |  |  |  |  |  |  |  |  |  |  |  |  |  |
| PhD training |  |  |  |  |  |  |  |  |  |  |  |  |  |  |  |  |
| Report/paper writing |  |  |  |  |  |  |  |  |  |  |  |  |  |  |  |  |
| Information to health authorities |  |  |  |  |  |  |  |  |  |  |  |  |  |  |  |  |

**REFERENCES**

1. Schuftan, C. A story to be shared: the successful fight against malaria in Vietnam. WHO WPRO and the global Roll Back Malaria Program. 2000. http://www.afronets.org
2. Phan VT. Epidemiological characteristics of malaria in Vietnam 1992-1997. http://www.ec-malaria.org/mcsi/book1_21.htm.

3. Cong LD. Results of malaria control in Vietnam 1992-1997 & malaria control plan for 1998-2000. http://www.ec-malaria.org/mcsi/book1_21.htm.

4. Phan VT. Epidemiologie du paludisme et lutte antipaludique au Vietnam. Hanoi: Editions Medicales Vietnam; 1998.

5. Erhart A, Thang ND, Toi LV, Hung LX, TuyTQ, Cong LD, Speybroeck N, Coosemans M, D'Alessandro U. Forest malaria in Vietnam: a challenge for control. Am J Trop Med Hyg 2003; *in press*.

6. TDR fifteenth programs report progress 1999-2000. http://www.who.int/tdr/research/progress9900/strategies/itm-malaria.htm. 2000. 31-10-2002.

1. WHO Expert committee on malaria. Twentieth report. http://mosquito.who.int/docs/ecr20_8.htm. 31-10-2002.
2. Combatting communicable diseases. Focus 2: Malaria, other vector-borne and parasitic diseases. <http://www.wpro.who.int/themes_focuses/theme1/focus2/t1f2cambodia.asp>. 8-7-0003.

**List of references published in the framework of the scientific collaboration between ITM and NIMPE, Hanoi:**

1. VERLE P., BINH L.N., LIEU T.T., YEN P.T., COOSEMANS M. (1996) ParaSight®-F test to diagnose malaria in hypo-endemic and epidemic prone regions of Vietnam. Tropical Medicine and International Health **1**, 794-796.
2. VERLE P, RUYEN NT, HUONG NT, BE NT, KONGS A, VAN DER STUYFT P, & COOSEMANS (1998) A simple field-test for detecting pyrethroids on impregnated nets. Tropical Medicine and International Health, **3**, 833-836.
3. VERLE P., LIEU T.T.T., KONGS A., Van der STUYFT P., & COOSEMANS M. (1999) Control of malaria vectors: Cost analysis in a province of northern Vietnam. Tropical Medicine and International Health **4 (**2) 139-145
4. VERLE P, TUY T.Q., KONGS A., COOSEMANS M. (1998) New challenges for malaria control in northern Vietnam. Research and review in Parasitology, **58**: 169-374
5. VAN BORTEL W, TRUNG HD, MANH ND, ROELANTS P, VERLE & COOSEMANS (1999) Identification of two species within the *Anopheles minimus* complex in northern Vietnam and their behavioural divergences. Tropical Medicine and International Health **4**, 257-265
6. Verlé P, Nhan DH, Tinh TT, Uylen TT, Thuong ND, Kongs A, Van der Stuyft P & Coosemans M (2000) Glucose-6-phosphate dehydogenase deficiency in northern Vietnam. Tropical Medicine and International Health **5**, 203-206
7. VAN BORTEL W, TRUNG HD, ROELANTS P, HARBACH RE, BACKELJAU Th & COOSEMANS M (2000) Molecular identification of *Anopheles minimus s.l*.: beyond distinguishing the members of species complex. Insect Molecular Biology **9**,335-340
8. Kengne P, Trung HD, BAIMAI V, Coosemans M & Manguin S (2001). A multiplex PCR-based method derived from random amplified polymorphic DNA (RAPD) markers for the identification of species of the *Anopheles minimus* group in Southeast Asia. Insect Molecular Biology,**10**, 425-435.
9. Van Bortel W, Harbach RE, Trung HD, Roelants P, Backeljau T & Coosemans M. (2001) Confirmation of *Anopheles varuna* in Vietnam previously misidentified and mistargeted as malaria vector *An.minimus*. American Journal of Tropical Medicine.**65**,729-732
10. MANGUIN S, MOUCHET J, COOSEMANS M. (2001) Identification moléculaire d’espèces jumelles d’anophèles : Exemple des complexes An. *minimus* et *An. dirus*, vecteurs majeurs du paludisme en Asie du Sud-Est. Med. Trop. 2001, **61**(6) :463-469.
11. Van Bortel W, SOCHANTA T, Harbach RE, SOCHEAT D, Roelants P, Backeljau T & Coosemans M. 2002 Presence of *Anopheles culicifacies* B in Cambodia established by PCR-RFLP assay developed for the identification of *Anopheles minimus* species A and C and four related species. Medical and Veterinary Entomology. **16**, 329-334.
12. MANGUIN S, KENGNE P., SONNIER L., HARBACH R.E., BAIMAI V., TRUNG H.D., COOSEMANS M. SCAR markers and multiplex PCR-based identification of isomorphic species in the *Anopheles dirus* complex of Southeast Asia. Med. Vet. Entomol. 2002, 16(1) : 46-54
13. Van Bortel W, Coosemans M (200) Suggesting new species? Reply on 'Evidence for a new species of *Anopheles minimus* from the Ryukyu archipelago, Japan' Journal of the American Mosquito Control Association.(in press)
14. Van Bortel W, TRUNG HD, Roelants P, Backeljau T & Coosemans M (2003). Population genetic structure of the malaria vector *Anopheles minimus* A in Vietnam. Heredity (in press).
15. Van Bortel W, Trung HD, Sochantha T, Keokenchanh K, Roelants P, Backeljau T, Coosemans M.  Eco-ethological heterogeneity of the members of the *Anopheles minimus* complex of Southeast Asia and its consequences on vector control. Journal of Medical Entomology (accepted)
16. Trung HD, Van Bortel W, Sochantha T, Keokenchanh K, Quang NT, Cong LD and Coosemans M. Malaria Transmission and Major Malaria Vectors in Different Geographical Areas of Southeast Asia. Tropical Medicine and International Health (submitted)
